# Risk Assessment and Toxic Effects of Metal Pollution in Two Cultured and Wild Fish Species from Highly Degraded Aquatic Habitats

**DOI:** 10.1007/s00244-013-9935-z

**Published:** 2013-07-11

**Authors:** Wael A. Omar, Khalid H. Zaghloul, Amr A. Abdel-Khalek, S. Abo-Hegab

**Affiliations:** 1 Department of Zoology, Faculty of Science, Cairo University, Giza, Egypt; 2 Department of Zoology, Faculty of Science, El-Fayoum University, El-Fayoum, Egypt

## Abstract

Lake Qaroun is an inland lake at the lowest part of El-Fayoum depression, Egypt. It receives agricultural and domestic non-treated drainage waters, which are also used for aquaculture in Qaroun area. The results of the present study aimed to provide comparable data between wild (collected from Lake Qaroun) and cultured (collected from Qaroun fish farms and the reference site) Nile tilapia *Oreochromis niloticus* and mullet *Mugil cephalus*, as indicators of natural and anthropogenic impacts on aquatic ecosystem as well as to evaluate the human hazard index associated with fish consumption. Metal concentrations in fish tissues showed a species-specific bioaccumulation pattern. Statistically significant differences were observed in the mean metal concentrations with lower bioavailability in *M. cephalus* compared with *O. niloticus* in internal vital organs (liver, kidney, and muscle) but much higher in external organs (gill and skin). Histopathological alterations and evident damages were observed in gill, liver, and kidney of both species collected from Lake Qaroun and Qaroun fish farms compared with those from the reference site. The results showed significant increase of plasma aspartate aminotransferase and alanine aminotransferase activity as well as creatinine and uric acid concentration in both fish species from polluted locations. The human health hazard index showed that the cumulative risk greatly increases with increasing fish consumption rate, thus yielding an alarming concern for consumer health.

The aquatic environment makes up the major part of our environment and resources. Therefore, its safety is directly related to human health. The excessive contamination of aquatic ecosystems has evoked major environmental and health concerns worldwide (McNeil and Fredberg 2011) because the aquatic environment is the ultimate recipient of pollutants produced by natural and anthropogenic sources (Cavas 2008).

Lake Qaroun, Egypt, witnessed several drastic changes affecting its role as an economic potential site for natural resources (Dardir and Wali 2009). It receives agricultural and domestic drainage water from El-Fayoum province which is loaded with heavy metals through a system of drainage canals that pass ~450 million cubic meters of wastewater annually to the lake, which greatly affect its biota (El-Shabrawy and Belmonte 2004; Fathi and Flower 2005). The fisheries of Lake Qaroun are already overexploited. The local fishermen are complaining of a serious decrease in fisheries (Gupta and Abd El-Hamid 2003). As a result, the Egyptian government has motivated the development of aquaculture and intensification of culture methods along the banks of Lake Qaroun especially for *Oreochromis niloticus* and *Mugil cephalus*. However, due to the regulation rules for use of water resources, the fish farms are allowed to use only water from the agricultural drainage network that finally discharge into the lake (Konsowa 2007).

The agricultural runoff has had cumulative negative impacts on the water quality, which is considered the main environmental factor controlling the state of health and disease in both cultured and wild fish (Mansour and Sidky 2002; Fathi and Flower 2005). Among the various toxic pollutants, heavy metals represent an interesting group of elements due to their strong impact on the stability of aquatic ecosystems, bioaccumulation in living organisms, and toxicity persistence (Has-Schon et al. 2006; De et al. 2010).

The present study area is considered ideal to evaluate the role of a normal agricultural community as a source of anthropogenic impact on aquatic ecosystems with a single source of water input from the River Nile and a single source of water output at Lake Qaroun, which lie only 30 km apart. This approach aims to provide comparable data between cultured and wild Nile tilapia *O. niloticus* and mullet *M. cephalus* collected from Lake Qaroun and fish farms around it compared with fish from a reference site irrigated with freshwater from the River Nile. In addition, the aim of our study was to evaluate human hazard indices associated with consuming fish from these degraded aquatic habitats.

## Materials and Methods

### Sites of Collection

**Fig. 1:**
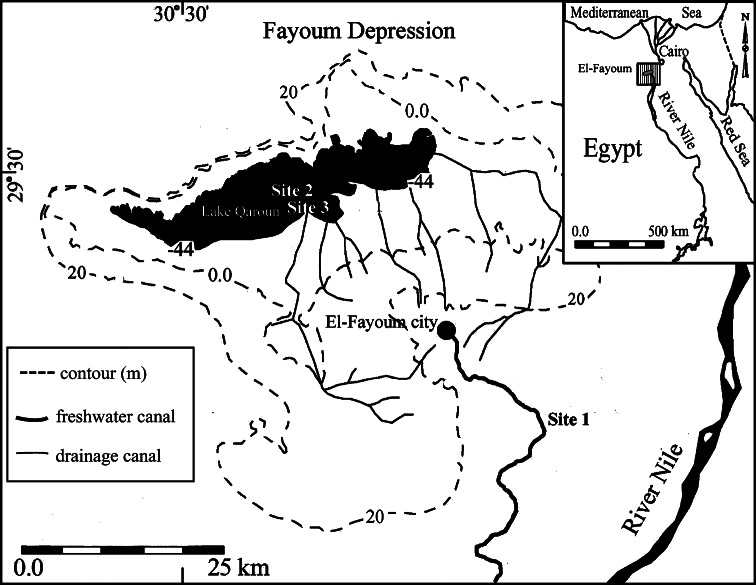
Map of El-Fayoum province showing the study sites

A total number of 48 adult fish of both species (16 fish/site) with average body length 17.56 ± 0.27 and 24.54 ± 0.67 cm and average body weight 103.66 ± 5.25 and 133.08 ± 4.81 g for *O. niloticus* and *M. cephalus*, respectively, were collected with the help of local fishermen from the following sites (Fig. 1):

- Site 1 (reference site): Fish farm of the Faculty of Agriculture, El-Fayoum University. irrigated with a branch of the River Nile. Located at global positioning system (GPS) reading of 29°17′45.19″N and 30°54′57.52″E.
- Site 2: The southwest side of Lake Qaroun at the outlet of El-Wadi drainage canal, which is one of the main drainage canals in El-Fayoum province. This site represents the wild habitat for the studied fish species. Located at GPS reading of 29°27′25.76″N and 30°38′51.53″E.
- Site 3: Four fish farms at the southern banks of Lake Qaroun. These farms depend on agricultural drainage water as a water source. Located at GPS reading of 29°26′43.52″N and 30°39′49.12″E.

The studied fish farms had nearly the same rearing conditions (polyculture of Nile tilapia and mullet), commercial formulated diet (25–35 % crude protein) and fish-stocking density (5–8 and 2–3 fish/m^3^ for Nile tilapia and mullet, respectively).

### Determination of Residual Heavy Metals

Residual heavy metals (copper [Cu], zinc [Zn], lead [Pb], iron [Fe], and manganese [Mn]) were measured using a flame atomic absorption spectrophotometer (Perkin Elmer-2280, USA) in fish tissues (liver, kidney, gill, skin, and muscle) according to the APHA (2005). Fish tissue samples were dried, acid-digested, and diluted with deionized water to known volume using the dry-ashing procedure proposed by Issac and Kerber (1971) and by Hseu (2004). Analytical blanks were run in the same way as the samples, and concentrations were determined using standard solutions prepared in the same acid matrix. Standards for instrument calibration were prepared on the basis of monoelement-certified reference solution inductively coupled plasma standard (Sigma-Aldrich). Standard reference material (Lake Superior fish 1946; National Institute of Standards and Technology [NIST], USA) was used to validate analysis, and the metal recoveries were between 90 and 110 %.

### Blood Sampling and Biochemical Measurements

Blood samples were withdrawn from the caudal vein of both studied fish species using sodium citrate as anticoagulant. Blood samples were centrifuged to obtain plasma for the determination of aspartate aminotransferase (AST; EC.2.6.1.1) and alanine aminotransferase activity (ALT; EC. 2.6.1.2; Reitman and Frankel 1957) as well as creatinine (Henry et al. 1974) and uric acid concentration (Barham and Trinder 1972) using enzymatic-colorimetric methods by means of commercial kits (Biodiagnostic, Egypt).

### Histological Studies

Fish gill, liver, and kidney were preserved in Bouin’s fixative. Tissues were processed, sectioned at 41 μm, and then stained using hematoxylin and eosin (Bernet et al. 1999).

### Statistical Analyses

Data were statistically analyzed using Student *t* test, analyses of variance (*F* test), and Duncan’s multiple range test to evaluate difference in means as indicated by different case letters in descending order, A, B, and C, at *P* *<* 0.05 using SAS version 9.1 (SAS 2006).

### Human Risk Assessment

The current risk assessment was determined according to United States Environmental Protection Agency (USEPA 2000). To calculate the level of exposure resulting from the consumption of a particular heavy metal in fish edible tissues (muscles and skin in case of Egyptian consumers), the equation for the average daily dose (ADD; average daily intake of a specific chemical over a lifetime]) was calculated as follows:

$${\text{ADD}}\left( {{\text{mg}}/{\text{kg}}/{\text{d}}} \right) = \left( {{\text{C}} \times {\text{IR}} \times {\text{EF}} \times {\text{ED}}} \right)/\left( {{\text{BW}} \times {\text{AT}}} \right)$$

where C is the mean total heavy-metal concentration in fish edible tissues (mg/kg), IR is the mean ingestion rate (0.0312 and 0.1424 kg/day for normal adults and habitual fish eaters, respectively), EF is the exposure frequency (365 days/year), ED is the exposure duration over a lifetime (70 years), BW is the body weight (70 kg for normal adults), and AT is the average life time (70 years × 365 days/year).

Risk assessment was quantified by calculating the hazard index (HI), which is the noncancer index of adverse health effects from intake of heavy metal in food, and it is expressed as the ratio of the ADD to the oral reference dose (RfD) of the heavy metal of concern according to the following equation:

$${\text{Hazard Index}} = {\text{ADD}}/{\text{Oral RfD}},$$

where oral RfD is the oral reference dose of heavy metal (mg/kg/days) based on the upper level of metal intake for an adult human with average body weight of 70 kg. The oral RfD for Cu, Zn, Pb, Fe, and Mn suggested by the United States Food and Agricultural Organization (FAO)/World Health Organization (WHO) (2006) and by WHO (2008) is 0.14, 0.214, 0.00357, 0.643, and 0.157 mg/kg/days, respectively. HI values <1.0 indicate that adverse health effects are not likely to occur. However, if the ADD of certain heavy metal exceeds its oral RfD and thus the HI is ≥1.0, it may be presumed that adverse health effects are expected to occur.

## Results

### Residual Heavy Metals in Fish Tissues

Concentrations of all analyzed heavy metals were significantly higher in fish tissues from sites 2 and 3 compared with those from the reference site (Tables 1, 2, 3, 4 and 5). Values of residual heavy metals in different fish tissues showed various degrees of species- and tissue-specific bioaccumulation pattern. Generally, the bioaccumulation of metals in *M. cephalus* was lower compared with *O. niloticus* in all studied internal vital organs (liver, kidney, and muscle) but much greater in external organs (gill and skin).

**Table 1:** Residual Cu concentrations (mg/kg dry weight [mean ± SE]) in selected organs of *O. niloticus* (*O. n.*) and *M. cephalus* (*M. c.*) (*N* = 8)

Organs		Site 1 (reference site)	Site 2 (Lake Qaroun)	Site 3 (Qaroun fish farms)	*P*_*F*_<
Liver	*O. n.*	2.30^a^ ± 0.23^C^	184.85^a^ ± 23.75^A^	111.31^a^ ± 3.40^B^	0.01
	*M.c.*	1.85^a^ ± 0.23^C^	68.84^b^ ± 6.60^B^	98.16^b^ ± 4.55^A^	0.01
	*P*_*t*_<	NS	0.01	0.05	
Kidney	*O. n.*	2.24^a^ ± 0.30^B^	50.74^a^ ± 4.88^A^	58.55^a^ ± 4.33^A^	0.01
	*M.c.*	1.42^b^ ± 0.17^C^	36.15^a^ ± 5.23^A^	17.08^b^ ± 1.45^B^	0.01
	*P*_*t*_<	0.05	NS	0.01	
Gill	*O. n.*	4.05^a^ ± 0.20^C^	14.92^a^ ± 1.26^A^	11.36^b^ ± 0.94^B^	0.01
	*M.c.*	2.75^b^ ± 0.27^C^	12.97^a^ ± 0.70^B^	15.67^a^ ± 0.86^A^	0.01
	*P*_*t*_<	0.01	NS	0.01	
Skin	*O. n.*	4.35^a^ ± 0.54^C^	10.83^a^ ± 0.68^A^	6.73^b^ ± 0.34^B^	0.01
	*M.c.*	3.71^a^ ± 0.48^B^	9.13^a^ ± 0.66^A^	8.69^a^ ± 0.41^A^	0.01
	*P*_*t*_<	NS	NS	0.01	
Muscle	*O. n.*	0.66^a^ ± 0.07^C^	13.22^a^ ± 1.50^A^	9.38^a^ ± 0.92^B^	0.01
	*M.c.*	0.29^b^ ± 0.04^B^	7.32^b^ ± 0.92^A^	6.05^b^ ± 0.45^A^	0.01
	*P*_*t*_<	0.01	0.01	0.05	

Means with the same superscript lower-case letter in the same column, and means with the same superscript capital letter in the same row, for each parameter are not significantly different. Student *t* test was performed between two fish species for each parameter in each site (*P*_*t*_), whereas *F* test was performed between three locations for each organ and each fish species (*P*_*F*_)

**Table 2:** Residual Zn concentrations (mg/kg dry weight [mean ± SE]) in selected organs of *O. niloticus* (*O. n.*) and *M. cephalus* (*M. c.*) (*N* = 8)

Organs		Site 1 (Reference site)	Site 2 (Lake Qaroun)	Site 3 (Qaroun fish farms)	*P*_*F*_<
Liver	*O. n.*	17.79^a^ ± 1.13^C^	114.77^a^ ± 3.24^A^	87.00^a^ ± 3.01^B^	0.01
	*M.c.*	13.04^b^ ± 0.85^C^	94.89^b^ ± 8.23^A^	59.57^b^ ± 4.79^B^	0.01
	*P*_*t*_<	0.01	0.05	0.01	
Kidney	*O. n.*	48.70^a^ ± 3.42^C^	196.31^a^ ± 7.48^A^	157.55^a^ ± 9.13^B^	0.01
	*M.c.*	35.66^b^ ± 2.32^C^	129.04^b^ ± 16.88^A^	80.59^b^ ± 7.87^B^	0.01
	*P*_*t*_<	0.01	0.01	0.01	
Gill	*O. n.*	12.95^a^ ± 0.95^B^	104.39^a^ ± 3.96^A^	107.57^a^ ± 3.94^A^	0.01
	*M.c.*	11.54^a^ ± 0.58^B^	127.67^b^ ± 3.08^A^	120.54^a^ ± 4.90^A^	0.01
	*P*_*t*_<	NS	0.01	NS	
Skin	*O. n.*	11.34^a^ ± 0.82^C^	83.83^a^ ± 7.45^B^	100.49^a^ ± 6.73^A^	0.01
	*M.c.*	8.62^b^ ± 0.72^C^	86.63^a^ ± 6.32^B^	101.55^a^ ± 4.23^A^	0.01
	*P*_*t*_<	0.05	NS	NS	
Muscle	*O. n.*	12.54^a^ ± 0.74^B^	39.95^a^ ± 1.74^A^	40.56^a^ ± 3.19^A^	0.01
	*M.c.*	8.92^b^ ± 0.39^C^	29.19^b^ ± 3.84^A^	22.36^b^ ± 1.08^B^	0.01
	*P*_*t*_<	0.01	0.05	0.01	

Means with the same superscript lower-case letter in the same column, and means with the same superscript capital letter in the same row, for each parameter are not significantly different. Student *t* test was performed between two fish species for each parameter in each site (*P*_*t*_), whereas *F* test was performed between three locations for each organ and each fish species (*P*_*F*_)

**Table 3:** Residual Pb concentrations (mg/kg dry weight [mean ± SE]) in selected organs of *O. niloticus* (*O. n.*) and *M. cephalus* (*M. c.*) (*N* = 8)

Organs		Site 1 (Reference site)	Site 2 (Lake Qaroun)	Site 3 (Qaroun fish farms)	*P*_*F*_<
Liver	*O. n.*	1.60^a^ ± 0.15^C^	36.40^a^ ± 2.49^A^	12.75^a^ ± 0.76^B^	0.01
	*M. c.*	1.02^b^ ± 0.09^B^	9.86^b^ ± 0.79^A^	9.23^b^ ± 1.15^A^	0.01
	*P*_*t*_<	0.01	0.01	0.05	
Kidney	*O. n.*	1.27^a^ ± 0.17^C^	68.44^a^ ± 5.26^A^	50.61^a^ ± 5.39^B^	0.01
	*M. c.*	0.84^b^ ± 0.06^B^	25.15^b^ ± 1.94^A^	29.58^b^ ± 4.13^A^	0.01
	*P*_*t*_<	0.05	0.01	0.01	
Gill	*O. n.*	0.85^a^ ± 0.07^C^	11.96^b^ ± 0.97^B^	17.36^b^ ± 2.29^A^	0.01
	*M. c.*	0.53^b^ ± 0.07^C^	14.25^a^ ± 0.33^B^	24.55^a^ ± 2.02^A^	0.01
	*P*_*t*_<	0.01	0.05	0.05	
Skin	*O. n.*	1.34^a^ ± 0.17^B^	14.81^a^ ± 1.21^A^	16.17^a^ ± 1.48^A^	0.01
	*M. c.*	0.48^b^ ± 0.08^B^	8.75^b^ ± 1.24^A^	10.50^b^ ± 0.79^A^	0.01
	*P*_*t*_<	0.01	0.01	0.01	
Muscle	*O. n.*	0.20^a^ ± 0.02^B^	3.76^a^ ± 0.98^A^	2.38^a^ ± 0.47^A^	0.01
	*M. c.*	0.27^a^ ± 0.03^B^	2.25^a^ ± 0.12^A^	1.77^a^ ± 0.25^A^	0.01
	*P*_*t*_<	NS	NS	NS	

Means with the same superscript lower-case letter in the same column, and means with the same superscript capital letter in the same row, for each parameter are not significantly different. Student *t* test was performed between two fish species for each parameter in each site (*P*_*t*_), whereas *F* test was performed between three locations for each organ and each fish species (*P*_*F*_)

**Table 4:** Residual Fe concentrations (mg/kg dry weight [mean ± SE]) in selected organs of *O. niloticus* (*O. n.*) and *M. cephalus* (*M. c.*) (*N* = 8)

Organs		Site 1 (reference site)	Site 2 (Lake Qaroun)	Site 3 (Qaroun fish farms)	*P*_*F*_<
Liver	*O. n.*	5.41^a^ ± 0.32^B^	142.55^a^ ± 12.80^A^	128.93^a^ ± 12.34^A^	0.01
	*M. c.*	3.49^b^ ± 0.32^B^	98.44^b^ ± 6.53^A^	99.82^a^ ± 7.46^A^	0.01
	*P*_*t*_<	0.01	0.01	NS	
Kidney	*O. n.*	3.36^a^ ± 0.37^B^	129.59^a^ ± 14.75^A^	127.31^a^ ± 3.82^A^	0.01
	*M. c.*	1.63^b^ ± 0.16^B^	106.43^a^ ± 12.69^A^	100.01^b^ ± 4.84^A^	0.01
	*P*_*t*_<	0.01	NS	0.01	
Gill	*O. n.*	3.42^a^ ± 0.54^B^	41.72^b^ ± 3.53^A^	35.92^b^ ± 1.82^A^	0.01
	*M. c.*	4.20^a^ ± 0.31^B^	58.41^a^ ± 3.31^A^	64.83^a^ ± 5.59^A^	0.01
	*P*_*t*_<	NS	0.01	0.01	
Skin	*O. n.*	6.98^a^ ± 0.29^B^	135.10^a^ ± 8.16^A^	121.14^a^ ± 5.64^A^	0.01
	*M. c.*	3.52^b^ ± 0.34^C^	120.34^a^ ± 6.03^A^	100.63^b^ ± 2.76^B^	0.01
	*P*_*t*_<	0.01	NS	0.01	
Muscle	*O. n.*	1.28^a^ ± 0.09^B^	12.05^a^ ± 1.94^A^	15.28^a^ ± 0.98^A^	0.01
	*M. c.*	1.31^a^ ± 0.16^C^	6.48^b^ ± 0.53^B^	10.87^b^ ± 0.87^A^	0.01
	*P*_*t*_<	NS	0.05	0.01	

Means with the same superscript lower-case letter in the same column, and means with the same superscript capital letter in the same row, for each parameter are not significantly different. Student *t* test was performed between two fish species for each parameter in each site (*P*_*t*_), whereas *F* test was performed between three locations for each organ and each fish species (*P*_*F*_)

**Table 5:** Residual Mn concentrations (mg/kg dry weight [mean ± SE]) in selected organs of *O. niloticus* (*O. n.*) and *M. cephalus* (*M. c.*) (*N* = 8)

Organs		Site 1 (Reference site)	Site 2 (Lake Qaroun)	Site 3 (Qaroun fish farms)	*P*_*F*_<
Liver	*O. n.*	1.87^a^ ± 0.31^C^	8.20^a^ ± 0.32^A^	5.85^a^ ± 0.46^B^	0.01
	*M. c.*	1.37^a^ ± 0.10^B^	4.42^b^ ± 0.38^A^	5.07^a^ ± 0.46^A^	0.01
	*P*_*t*_<	NS	0.01	NS	
Kidney	*O. n.*	1.55^b^ ± 0.22^B^	19.05^a^ ± 1.58^A^	15.92^a^ ± 1.32^A^	0.01
	*M. c.*	2.29^a^ ± 0.08^B^	16.78^a^ ± 3.15^A^	6.82^b^ ± 0.25^B^	0.01
	*P*_*t*_<	0.01	NS	0.01	
Gill	*O. n.*	1.00^a^ ± 0.04^B^	10.99^b^ ± 0.42^A^	11.70^b^ ± 0.59^A^	0.01
	*M. c.*	0.29^b^ ± 0.05^C^	31.44^a^ ± 4.61^A^	17.72^a^ ± 1.21^B^	0.01
	*P*_*t*_<	0.01	0.01	0.01	
Skin	*O. n.*	1.00^a^ ± 0.03^B^	10.17^a^ ± 0.41^A^	9.49^b^ ± 0.20^A^	0.01
	*M. c.*	1.07^a^ ± 0.20^B^	11.53^a^ ± 1.02^A^	12.55^a^ ± 1.03^A^	0.01
	*P*_*t*_<	NS	NS	0.05	
Muscle	*O. n.*	0.89^a^ ± 0.08^B^	1.81^a^ ± 0.41^A^/B	2.22^a^ ± 0.33^A^	0.05
	*M. c.*	0.71^a^ ± 0.11^B^	1.13^a^ ± 0.26^B^	1.82^a^ ± 0.23^A^	0.05
	*P*_*t*_<	NS	NS	NS	

Means with the same superscript lower-case letter in the same column, and means with the same superscript capital letter in the same row, for each parameter are not significantly different. Student *t* test was performed between two fish species for each parameter in each site (*P*_*t*_), whereas *F* test was performed between three locations for each organ and each fish species (*P*_*F*_)

### Blood Biochemical Parameters

Plasma AST and ALT activities were used as indicative parameters of liver function, whereas plasma creatinine and uric acid were used as indicative parameters of kidney function. The highest values were always recorded in wild fish from Lake Qaroun and the lowest values in cultured fish from reference site (Table 6).

**Table 6:** Indicative biochemical parameters of liver and kidney functions of *O. niloticus* (*O. n.*) and *M. cephalus* (*M. c.*), mean ± SE, *N* = 8

Organs		Site 1 (Reference site)	Site 2 (Lake Qaroun)	Site 3 (Qaroun fish farms)	*P*_*F*_<
AST (U/l)	*O. n.*	61.50^a^ ± 6.00^B^	114.87^a^ ± 2.04^A^	107.00^a^ ± 6.01^A^	0.01
	*M. c.*	44.12^b^ ± 1.86^C^	100.75^a^ ± 6.16^A^	87.50^b^ ± 0.62^B^	0.01
	*P*_*t*_<	0.05	NS	0.01	
ALT (U/l)	*O. n.*	40.75^a^ ± 4.10^C^	87.62^a^ ± 3.05^A^	77.00^a^ ± 3.49^B^	0.01
	*M. c.*	26.50^b^ ± 1.65^C^	74.50^b^ ± 4.63^A^	39.50^b^ ± 2.39^B^	0.01
	*P*_*t*_<	0.01	0.05	0.01	
Creatinine (mg/dl)	*O. n.*	1.74^a^ ± 0.13^C^	4.13^a^ ± 0.22^A^	3.12^a^ ± 0.11^B^	0.01
	*M. c.*	1.28^b^ ± 0.09^C^	3.08^b^ ± 0.20^A^	2.41^b^ ± 0.23^B^	0.01
	*P*_*t*_<	0.05	0.01	0.05	
Uric acid (mg/dl)	*O. n.*	8.64^a^ ± 0.27^C^	32.75^a^ ± 0.55^A^	27.23^a^ ± 1.31^B^	0.01
	*M. c.*	5.92^b^ ± 0.42^C^	28.62^b^ ± 1.38^A^	17.26^b^ ± 0.33^B^	0.01
	*P*_*t*_<	0.01	0.05	0.01	

Means with the same superscript lower-case letter in the same column, and means with the same superscript capital letter in the same row, for each parameter are not significantly different. Student *t* test was performed between two fish species for each parameter in each site (*P*_*t*_), whereas *F* test was performed between three locations for each biochemical parameter and each fish species (*P*_*F*_)

### Histological Studies

**Fig. 2:**
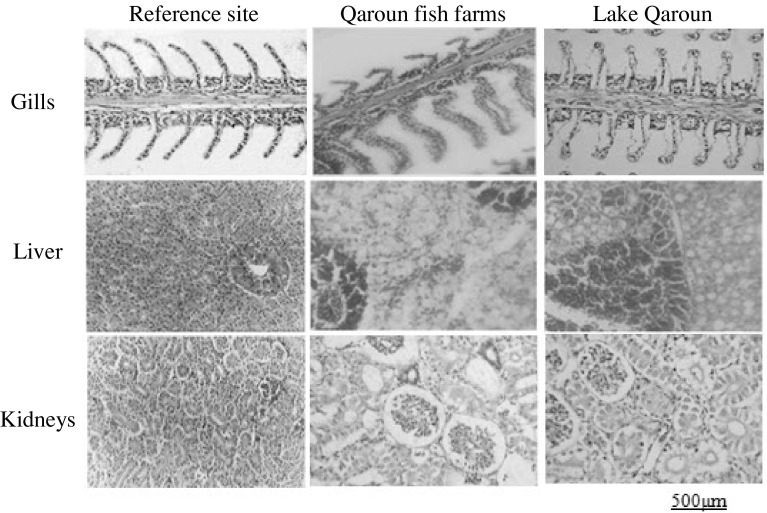
Histological sections of *O. niloticus* tissues

**Fig. 3:**
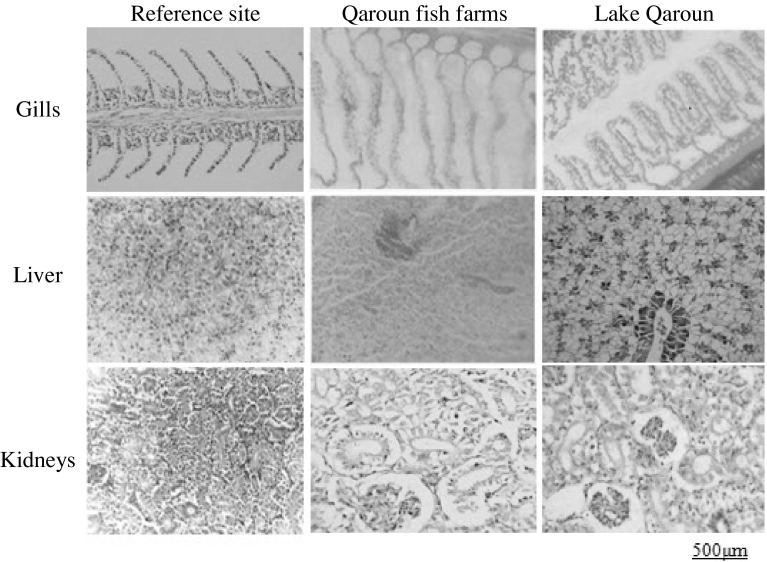
Histological sections of *M. cephelus* tissues

Sections of gill, liver and kidney of both studied fish species collected from the reference site showed normal histological structure. However, histopathological alterations and evident damage were obvious in gill, liver, and kidney of *O. niloticus* and *M. cephalus* collected from the other sites (Figs. 2 and 3).

#### Gill

Gill sections of both fish species from the reference site showed well-structured primary filaments and secondary lamellae with flat epithelial cells and chloride cells located at the bases of the secondary lamellae. Evident histopathological changes appeared in samples from the polluted sites. They generally included hyperplasia, degenerative changes, congestion in the lamellar blood vessels, clavate lamellae formation, ballooning degeneration and desquamation of the epithelium, oedema with epithelial lifting at the base of secondary lamellae, and intense rupture and peeling of lamellar epithelia.

#### Liver

Liver sections of both fish species from the reference site showed normal structure with compactly arranged hepatocytes. Sinusoids were scattered randomly all over the hepatocytes. Samples of the other sites showed marked deterioration in liver histoarchitecture, including infiltration of red blood cells through the hepatocytes with congestion, dilated intercellular spaces, peliosis (the replacement of liver tissue with blood-filled cavities without an endothelial cell lining), cloudy swelling, vacuolar degeneration, and tissue disorientation with rupture of parenchyma cells and single-cell necrosis.

#### Kidney

Kidney sections of both fish species from the reference site showed uniformly functional tubules and normal hematopoietic tissue in the interstices of the tubules. Kidney sections from polluted sites declared a progressive damage of kidney tubules associated with tissue disorientation, tubular necrosis, separation of tubular epithelial cells with peritubular oedema, decreased intertubular spaces, cloudy swelling of renal tubules, and irregularities including vacuolar degradation.

### Human Risk Assessment

Cu, Zn, and Pb concentrations were greater than the upper level of intake in food for human consumption (10, 15 to 20, and 0.25 mg/days, respectively) according to WHO (2008) in both fish species collected from sites 2 and 3. Meanwhile, values of Fe and Mn were within safe levels or human consumption (45 and 11 mg/days, respectively) according to FAO/WHO (2006) and WHO (2008) in all study sites. Table 7 shows that HI values for muscle consumption were <1.0 for all studied metals, except in the case of Pb, at greater subsistence rate of fish consumption from both Lake Qaroun and Qaroun fish farms. Meanwhile, HI values for skin consumption were <1.0 for all studied metals, except in case of Pb, at both rates of consumption (mean ingestion rate and greater subsistence rate for habitual fish consumers). Values of HI calculated for muscle and skin samples of fish collected from the reference site were within safe limits at both rates of consumption. Generally, risk values for analyzed heavy metals do not pose unacceptable risks at mean ingestion rate for muscles, but the risk greatly increases for muscles and skin, either separately or collectively, when fish are consumed at a greater rate.

**Table 7:** HI for muscle and skin consumption of *O. niloticus* (*O. n.*) and *M. cephalus* (*M. c.*) calculated at mean ingestion and subsistence ingestion rates

		Site 1 (Reference site)		Site 2 (Lake Qaroun)		Site 3 (Qaroun fish farms)	
		HI_m_	HI_s_	HI_m_	HI_s_	HI_m_	HI_s_
Copper	*O. n.* ^a^	0.002	0.014	0.043	0.036	0.029	0.021
	*O. n.* ^b^	0.009	0.063	0.193	0.157	0.136	0.100
	*M. c.* ^a^	0.001	0.012	0.021	0.029	0.021	0.029
	*M. c.* ^b^	0.004	0.054	0.107	0.136	0.086	0.129
Zinc	*O. n.* ^a^	0.026	0.024	0.084	0.173	0.084	0.210
	*O. n.* ^b^	0.119	0.108	0.379	0.799	0.383	0.953
	*M. c.* ^a^	0.019	0.018	0.061	0.182	0.047	0.210
	*M. c.* ^b^	0.085	0.082	0.276	0.822	0.210	0.967
Lead	*O. n.* ^a^	0.028	0.167	0.560	1.961^c^	0.280	1.961^c^
	*O. n.* ^b^	0.112	0.756	2.241^c^	8.403^c^	1.401^c^	9.244^c^
	*M. c.* ^a^	0.028	0.056	0.280	1.120^c^	0.280	1.401^c^
	*M. c.* ^b^	0.140	0.280	1.401^c^	5.042^c^	1.121^c^	5.882^c^
Iron	*O. n.* ^a^	0.001	0.005	0.008	0.093	0.011	0.084
	*O. n.* ^b^	0.004	0.022	0.039	0.428	0.048	0.383
	*M. c.* ^a^	0.001	0.003	0.005	0.084	0.008	0.070
	*M. c.* ^b^	0.004	0.011	0.020	0.381	0.034	0.319
Manganese	*O. n.* ^a^	0.003	0.003	0.006	0.032	0.006	0.025
	*O. n.* ^b^	0.011	0.013	0.025	0.134	0.032	0.121
	*M. c.* ^a^	0.002	0.003	0.006	0.032	0.006	0.038
	*M. c.* ^b^	0.009	0.014	0.013	0.146	0.025	0.166

*HI*_*m*_ HI for muscle, *HI*_*s*_ HI for skin

^a^0.0312 kg/day (mean ingestion rate)

^b^0.1424 kg/day (subsistence ingestion rate)

^c^HI > 1, which is the point at which adverse health effects are expected to occur

## Discussion

Aquaculture is currently the largest single source of fish supply in Egypt accounting for almost 65 % of the total fish production of the country. Most aquaculture production in Egypt depends on freshwater or brackish water species in the Nile delta region with tilapia, mullet, and carp making up more than 97 % of the total country production in 2007 (GFCM 2010), thus indicating that tilapia and mullet in particular are widely accepted by Egyptian consumers.

Fish have been widely used as bioindicators of metal pollution (Evans et al. 1993). Significant quantities of heavy metals are discharged into aquatic environments, which can be strongly accumulated and biomagnified along the aquatic food chains, thus resulting in sublethal effects or death in local fish populations (Almeida et al. 2002; Xu et al. 2004). Bioaccumulation of trace metals in fish is dependent on both the bioavailable concentration and species-specific physiological and ecological characteristics. Metal distribution between the different tissues within an organism depends on the mode of exposure and can serve as a pollution indicator (Maheswari et al. 2006). The bioaccumulation of metals is a useful tool for studying the biological role of the metals present at increased levels in fish as well as assessment of public health risk (Reinfelder et al. 1998).

The three sampling sites in the present study were selected to investigate the contribution of the whole community of El-Fayoum province to the pollution conditions in the area. Having in mind that the reference site lies east in close proximity to the River Nile (the source of freshwater for the whole province) and that the other two sites lie west at the rear far end of the province where agricultural drainage water passes to the closed basin of Lake Qaroun, these sites ideal for the present study.

### Residual Heavy Metals

Among the myriad pollutants released into aquatic ecosystems, heavy metals have received considerable attention due to their toxicity, long-term persistence, bioaccumulation, and biomagnification at various trophic levels (Ololade et al. 2008). Fish may absorb dissolved elements and trace metals and then accumulate them in various tissues in significant amounts above those found in their environment, thus exhibiting elicited toxicological effects (McCarthy and Shugart 1990). Bioaccumulation of metals in tissues varies from metal to metal and differs in various organisms also among different organs of the same organism (Watanabe et al. 2003; Masoud et al. 2007). Moreover, the uptake of metals by fish involves transfer of metals through gill, intestine, or skin to the circulatory system and then proceeds to the organs of detoxification (liver, spleen, and kidney) either for long-term storage or excretion (Heath 1987). Moreover, Koca et al. (2005) postulated that the accumulation patterns of contaminants in fish and other aquatic organisms depend both on uptake and elimination rates of contaminants. Gills and skin are in direct contact with the aquatic medium; therefore, metal concentrations in these organs reflect their concentrations in the external environment. In contrast, the concentrations in liver and kidney represent the rates of bioaccumulation and detoxification of pollutants. Liver is the principal organ responsible for the detoxification, transformation, and storage of toxic materials and thus is an active site of pathological effects induced by contaminants (Zauke et al. 1999). The haemopoietic functions of liver and kidney with abundant blood supply explain their greater accumulation of Fe (Blasco et al. 1998). The high levels of Cu in liver tissue can be explained by its relation to low molecular–weight proteins (metallothionein-like), which are concentrated in hepatic tissues (Hamza-Chaffai et al. 1996). The lowest bioaccumulated heavy metals in muscles may be correlated with the fat-content in muscle tissues, low fat affinity to combine with heavy metals, and/or low metabolic activity of muscle (Uluturhan and Kucuksezgin 2007). Generally, the difference in bioaccumulation pattern among organs of both studied fish species may be attributed to the difference in feeding habits and lifestyle of both studied species. As indicated by Rejomon et al. (2010), metabolic requirements for specific trace metals in the individual species, or differences in dietary preferences, may account for the differences in metal accumulation among fish species.

### Indicative Parameters of Liver Functions

Enzymes, such as aminotransferases (AST and ALT), may increase in blood as a result of leakage from cells in injured tissues and hence are used as indicators of specific or multiple organ dysfunctions (Boyd 1983). Moreover, Boyd (1983) suggested that liver is rich in AST and ALT, so any damage could result in liberation of large quantities of these enzymes to the blood. AST and ALT are quantitatively important in transamination of amino acids, which represents one of the main pathways for amino acid synthesis and deamination, thereby allowing interplay between carbohydrate and protein metabolism during the fluctuating energy demands of the organism in various adaptive situations (Verma et al. 1981).

The detected increase in AST and ALT activities may be attributed to the damage in liver tissues by the action of bioaccumulated heavy metals, which are metabolized mainly by the hepatic parenchyma cells as an important detoxification defense mechanism against toxicants. The present findings are supported by Wu et al. (2003) because they recorded increase of both AST and ALT activities in stressed fish due to hepatic cells injury or increased synthesis of these enzymes by the liver.

### Indicative Parameters of Kidney Functions

Plasma creatinine and uric acid can be used as approximate indices of glomerular filtration rate and kidney dysfunction (Maita et al. 1984). Low levels of creatinine and uric acid have no significance, but their increase indicates several disturbances in kidney (Maxine and Benjamin 1985). The increase in plasma creatinine and uric acid in fish collected from Lake Qaroun and fish farms around it, compared with those from the reference site, may be attributed to the action of accumulated heavy metals on renal tubules that consequently caused pathological changes in kidney, which were confirmed in the histological examination. Moreover, the increased plasma creatinine values may be the consequence of glomerular insufficiency, increased muscle catabolism, and/or impairment in carbohydrate metabolism (Yang and Chen 2003), whereas the increased uric acid levels mainly refer to disturbance in kidney functions (Lockhart and Metner 1984).

### Histological Studies

Microscopic examination of target tissues is an important end point in the evaluation of toxic potential and risk assessment of chemicals in the environment (Velma and Tchounwou 2010) and the discrimination between different polluted sites (Dietze et al. 2001). Gills are directly affected by contaminants owing to their direct and continuous contact with the external medium and their functions in respiratory gas exchange, osmoregulation, excretion of nitrogenous waste, and acid-base regulation (Bhagwant and Elahee 2002). Toxic environmental conditions can result in several structural changes, which appear to be a compensatory mechanism to increase the epithelial thickness, thus preventing entry of toxic ions into the bloodstream or to compensate for osmotic imbalance (Velcheva et al. 2010). The observed lifting and swelling of the lamellar epithelium mainly develop with the purpose to increase the distance across which waterborne irritants can diffuse to reach the bloodstream (Mallatt 1985). Ballooning dilatation in gill filament is considered as an ion trap that acts to concentrate trace metals and favor cell adhesion between neighboring secondary lamellae (Bhagwant and Elahee 2002).

The liver receives particular attention in toxicological investigations of different fish species due to its high metabolic activity (Velcheva et al. 2010). Bogiswariy et al. (2008) reported that toxicant-exposed liver shows vacuolation because of the excessive accumulation of fat in cytoplasm. The detected vacuolar structures filled with cellular debris are thought to be a response of Kupffer cells (responsible for detoxification) to various pollutants. Moreover, the evident damage of the central vein, degeneration of liver tissues, and necrosis observed in samples from the polluted sites could be attributed to the accumulation and infiltration of neutrophils and lymphocytes as indicated by Koca et al. (2005). These histopathological changes suggest high metabolic activity in hepatocytes in response to the uptake of heavy metals (Thophon et al. 2003).

The observed signs of necrosis and disintegration of kidney tubules due to heavy-metal toxicity were reported in several fish species as described by Oliveira-Ribeiro et al. (2002) for arctic charr (*Salvelinus alpinus*), by Thophon et al. (2003) for white seabass (*Lates calcarifer*), and by Zaghloul et al. (2011) for sole (*Solea aegyptiaca*). These results indicate that heavy-metal contamination definitely affects structural and functional attributes of fish kidney. Moreover, degeneration of tubular epithelial cells and tubular necrosis may be due to the accumulation of inflammatory cells associated with metal toxicity (Velma and Tchounwou 2010).

The deterioration in histoarchitecture observed in gill, liver, and kidney of both fish species collected from Lake Qaroun and fish farms around it was generally in accordance with the results of residual heavy metals, which suggests too slow defense mechanisms in these tissues to immobilize or eliminate heavy metals and shows the sensitivity of fish cells to metal exposure.

### Human Risk Assessment

Fish are widely consumed in many parts of the world due to their high protein content, low saturated fat, and sufficient omega fatty acids with an omega-6 to omega-3 ratio favorable for human health (USEPA 2004). Therefore, the human health risk associated with heavy-metals poisoning and increased levels in marine and freshwater fish have resulted in worldwide advisories for fish consumption. Fish-consumption information is essential for assessing human health implications associated with the consumption of chemically contaminated fish (Copat et al. 2012). Therefore, a health assessment approach was performed to evaluate the current risk status associated with the consumption of heavy metal–contaminated fish at two different consumption rates: mean ingestion rate and greater subsistence rate. Despite the low health hazard values for each heavy metal separately, fish edible tissues contain totally abundant quantities of different heavy metals, which may lead to adverse health effects to humans, i.e., the cumulative risk effects of metals together give an alarming sign. Moreover, it indicates that the health of fish-dependent consumers is endangered around the studied polluted sites. Greater amounts of agricultural and domestic wastewaters discharged into the lake have a serious impact on its aquatic ecosystem as indicated by the effect of the surrounding agricultural community, which extends along an area not more than 30 km. In addition to the need to conduct a comprehensive study of the aquatic resources, there is also an urgent need to find a cost-effective method to decrease pollution in Lake Qaroun and the surrounding ecosystem.
